# 2,4,6-Trichloroanisole Off-Flavor Screening in Green *Coffea arabica* by a Novel Vocus NO^+^ CI-MS Method: A Study on Green Coffee from Different Geographical Origins

**DOI:** 10.1021/acs.jafc.2c03899

**Published:** 2022-08-30

**Authors:** Andrea Romano, Luciano Navarini, Valentina Lonzarich, Sara Bogialli, Paolo Pastore, Luca Cappellin

**Affiliations:** † Department of Chemical Sciences, University of Padua, Via Marzolo 1, 35131 Padua, Italy; ‡ Illycaffè spa, via Flavia 110, 34147 Trieste, Italy; § Aromalab, Illycaffè spa, Area Science Park, Padriciano 99, Trieste 34149, Italy

**Keywords:** coffee, Rio defect, 2,4,6-trichloroanisole (TCA), Vocus CI-MS

## Abstract

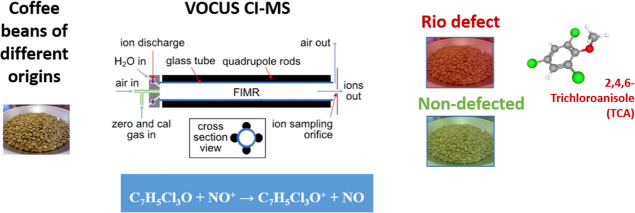

The Rio defect is a coffee off-flavor associated to unpleasant medicinal, phenolic, and iodine-like notes. 2,4,6-Trichloroanisole (TCA) is the main marker of this alteration. A new approach for TCA detection in green coffee beans was evaluated using chemical ionization time-of-flight mass spectrometry and employing a Vocus ion source and ion-molecule reactor (IMR). The sample set consisted of 22 green *Coffea arabica* from different geographical origins, four of which presented the Rio defect according to an expert cup-tasting panel. Vocus CI-MS was able to perform TCA detection in 3 s, with a sensitivity comparable to that of a sensory panel and showed remarkably good correlation (*R*^2^ ≥ 0.9997) with SPME–GC–MS measurements carried out on coffee headspace and hydro-alcoholic extracts. The results demonstrate how the introduction of new quick and sensitive analytical tools could help provide a more comprehensive picture of the Rio coffee off-flavor.

## Introduction

The Rio defect in coffee is associated to a strongly unpleasant aroma, characterized by medicinal, phenolic, and iodine-like notes.^1^ The Rio defect affects 20% of Brazilian coffees, but it has been reported in coffees from other geographical origins as well. Studies on coffee beans and brews presenting the Rio defect have highlighted 2,4,6-trichloroanisole (TCA) as the most likely marker of this sensory alteration.^2−4^ TCA was extracted from coffee by simultaneous distillation–extraction and analyzed—in either non-derivatized or derivatized forms—by means of GC–MS, and levels of 1–100 parts per billion (ppb or μg/L) of TCA were found in green coffee beans. TCA concentrations are reduced by approximately 50% following bean roasting, but this is not sufficient to remove the defect as the orthonasal and retronasal TCA perception thresholds in brewed coffees are 8 and 1–2 parts per trillion (ppt or ng/L), respectively.^3^ Analogous to what was observed for TCA in wine,^5^ TCA in coffee might be the result of enzymatic *o*-methylation of 2,4,6-trichlorophenol (TCP) catalyzed by several strains of filamentous fungi. In support of this hypothesis, high concentrations of TCP have been found together with TCA in Rio-tainted coffees.^4^ When non-tainted coffee samples were spiked with about 25 ppb TCA and submitted to a jury of coffee experts, the experts were able to positively recognize the Rio off-flavor.^3^

TCA is a well-known marker of sensory alteration in several food and beverage products. Perception thresholds from 0.03 to 1–2 and 4 ng/L are reported for water and white wine, respectively.^6^ The wine industry is the field where TCA is most studied: its origin has been demonstrated to be mostly related to cork stoppers^5^ with TCA alone being responsible for more than 80% of cork off-flavor problems.^7^ In wine, TCA reduces aroma perception even for contaminations below its sensory perception threshold,^8^ which can be explained considering that TCA affects aroma perception inhibiting ciliary transduction channels within the nasal mucosa.^9^ The level of TCA contamination in cork can be expressed as “releasable TCA”: the expression refers to the amount of TCA (in ng/L) that is released after soaking the contaminated corks in a hydroalcoholic solution or white wine.^10,11^ The TCA contained in the extracts is concentrated by means of solid-phase microextraction (SPME) or stir-bar sorptive extraction and finally analyzed by gas chromatography (GC) coupled to mass spectrometry (MS) or using an electron capture detector.^12^ Recently, a novel approach was tested for TCA determination in cork, based on chemical ionization time-of-flight mass spectrometry and employing a Vocus ion source and ion-molecule reactor (IMR).^13^ The technique allowed to perform 3 s TCA quantitation directly on whole cork stoppers and at concentration levels below the perception threshold. These characteristics rendered this approach suitable for direct monitoring of the bottling process, allowing to prevent the use of tainted cork stoppers.

A similar approach might be applicable to other food products affected by TCA contamination. In the present work, green coffee bean batches originating from different countries were analyzed. Some of them presented the Rio defect according to a panel of expert coffee cup tasters. Vocus CI-MS was used to perform a very fast (3 s/sample) TCA determination in coffee bean headspace, affording results in agreement with sensory analysis. Vocus CI-MS results on TCA presence and relative levels in the headspace of the contaminated coffees were also confirmed by GC–MS analysis, carried out according to two different methodologies.

## Materials and Methods

### Samples

A total of 22 green arabica (*Coffea arabica* L.) samples from five different geographical origins [Brazil (eight samples belonging to different batches), Colombia (2), Ethiopia (4), Guatemala (4), and Nicaragua (4)] were supplied and selected by illycaffè S.p.A. Quality Control Department (Trieste, Italy). The good-quality coffee samples (18 samples), all wet-processed with zero primary and secondary defects, were selected on the basis of standard procedures of sorting and visual aspect, moisture content, screen size, and cup quality. Four different Rio defective samples from Brazil were ad hoc-selected and provided by Experimental Agrícola do Brasil Ltda—São Paulo—SP, Brazil. The cup quality was evaluated by sensory analysis in a sensory laboratory designed in accordance with ISO 8589:2007 sensory analysis—general guidance for the design of test rooms. A panel composed of six trained experts performed the sensory assessment (descriptive profiling with a consensus vocabulary) of espresso brews in duplicate. The coffee samples were preliminarily roasted to a medium roasting degree (corresponding to a total weight loss equal to 15%, w/w) in a laboratory roaster (Probat, Germany), and the corresponding espresso coffee was brewed using a professional machine (La Marzocco, Italy) with a water temperature of 94 °C and a pressure of 9 bar, according to the espresso preparation standard as follows: 14.0 g of roasted coffee powder to obtain 50 mL (double shot) of beverage in 25 s, on one percolation group of the espresso machine. Samples with high scores and evaluated with no defects were used to constitute the set of “good-quality” green coffee samples.

### Sample Processing

Green bean samples (30 g) kept under liquid nitrogen were ground for 1 min by using an M20 Universal mill (IKA Werke GmbH, Germany).

### TCA Determination by NO^+^ CI-MS

**Figure 1:**
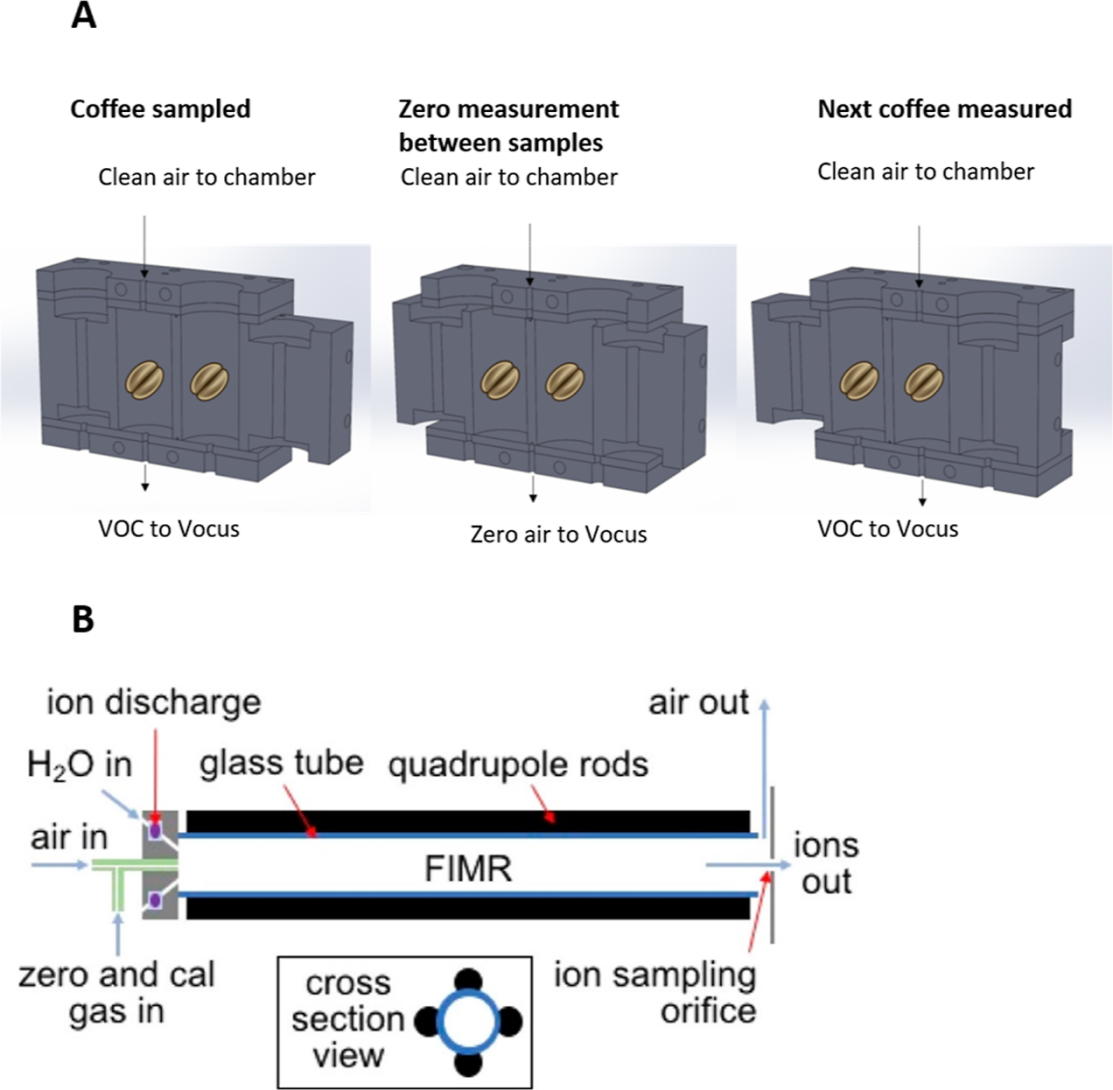
Schematic representation of the headspace sampling (A) and Vocus CI-MS (B). Vocus CI-MS reprinted with permission from ref (16). Copyright 2018 American Chemical Society.

The instrumentation used consisted of a Vocus 2R chemical ionization high-resolution mass spectrometer (Tofwerk AG, Switzerland), coupled to a custom headspace analysis device (Figure 1). The sampling device consisted of a preheating chamber and a sampling chamber, both constantly kept at 120 °C. The sample (5 g of ground green coffee powder) was first introduced into the preheating chamber and kept there for 2 min, constantly flushed with synthetic air at a flow of 1 L/min. The sample was then transferred into the sampling chamber (internal volume = 50 mL), which was also constantly flushed with synthetic air at a flow of 1 L/min. The synthetic air used was Alphagaz 1 air (Air Liquide, France), containing—according to the manufacturer’s specifications—≤3 ppm water vapor, ≤1 ppm CO, ≤1 ppm CO_2_, and ≤0.5 ppm total hydrocarbons as main impurities. The Vocus CI-MS sampling line (PTFE, *T* = 120 °C, 1/8 in. i.d.) was connected to the headspace sampler outlet and set at a flow of 150 mL/min. Under these conditions, the sample would very rapidly equilibrate with the headspace allowing for 3 s measurements, of which 1 s is settling time and 2 s is actual acquisition.

The Vocus 2R high-resolution chemical ionization mass spectrometer was equipped with a discharge reagent-ion source, operating at ≈2 mbar and generating NO^+^ reagent ions from synthetic air. TCA ions were produced with chemical ionization (CI) via charge transfer according to the following reaction
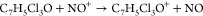


Fragmentation of the analyte ions was negligible in the selected setup. The Vocus 2R is also equipped with a focusing IMR consisting of a glass tube with resistive heating, mounted inside a radio frequency (RF) quadrupole. The RF field focuses ions to the central axis, improving detection efficiency of product ions. The IMR was operated at 1.5 mbar and 150 °C, and it was coupled to a time-of-flight mass analyzer. The mode of functioning of the VOCUS 2R is further detailed in the literature.^14−16^

A standard mixture (Carbagas, Switzerland) containing benzene, toluene, and xylene (10 ppm in pure nitrogen) was introduced at a flow of 5 mL/min into the sample flow in order to monitor the primary-ion stability. The signal intensity, expressed in counts per second (cps) of the spectral peaks at *m*/*z* 209.940 (corresponding to C_7_H_5_^35^Cl_3_O^+^), *m*/*z* 211.937 (C_7_H_5_^35^Cl_2_^37^ClO^+^), and *m*/*z* 213.934 (C_7_H_5_^35^Cl^37^Cl_2_O^+^), was summed and used as the signal for TCA. The benzene signal C_6_H_6_^+^ was used as the internal standard to correct for possible sensitivity drifts.

### Releasable TCA Measurement

TCA in green coffee was determined by SPME–GC–MS after hydro-alcoholic extraction using a procedure adapted from the ISO methodology for releasable TCA determination in cork stopper granules.^10^ 40 g of ground green coffee powder was placed in a 2 L flask and completely covered with 12% v/v hydro-alcoholic solution and soaked for 24 h. 10 mL of solution was transferred into a 20 mL vial and 3 g of NaCl and 100 μL of an internal standard solution consisting of 10 ng/L 2,4,6-trichloroanisole-*d*_5_ (TCA-*d*_5_) in a hydro-alcoholic solution, 12% v/v, were added. The vial was kept under stirring at 35 °C, and headspace volatile compounds were collected for 15 min using a SPME fiber coated with divinylbenzene/carboxen/polydimethylsiloxane (DBV/CAR/PDMS, Sigma-Aldrich, US). SPME fibers were desorbed at 260 °C for 2 min in the splitless mode in the injector port of a gas chromatograph interfaced with a mass detector (GC Agilent 7820A with Agilent 5977B MSD, Agilent Technologies, US). Separation was achieved on an Agilent HP-5 capillary column (30 m × 0.25 mm ID x 0.25 μm film thickness). The GC oven temperature program was: 35 °C for 6 min and then ramped to 280 °C at 15 °C/min and held at 280 °C for 5 min. Helium was used as carrier gas with a constant column flow rate of 1 mL/min. The mass detector was operated in the electron ionization mode (EI, internal ionization source; 70 eV) and in the single-ion monitoring (SIM) mode. Ions *m*/*z* 195, 210, and 212 were used for TCA and *m*/*z* 199, 215, and 217 for TCA-*d*_5_. Ions *m*/*z* 195 and 215 were employed for TCA and TCA-*d*_5_, respectively, and results are expressed in ng/L of releasable TCA. The set of calibration solutions for TCA was obtained by adding known concentrations of analytes to the hydro-alcoholic solution.

### TCA Measurement by Headspace-SPME GC–MS

**Figure 2:**
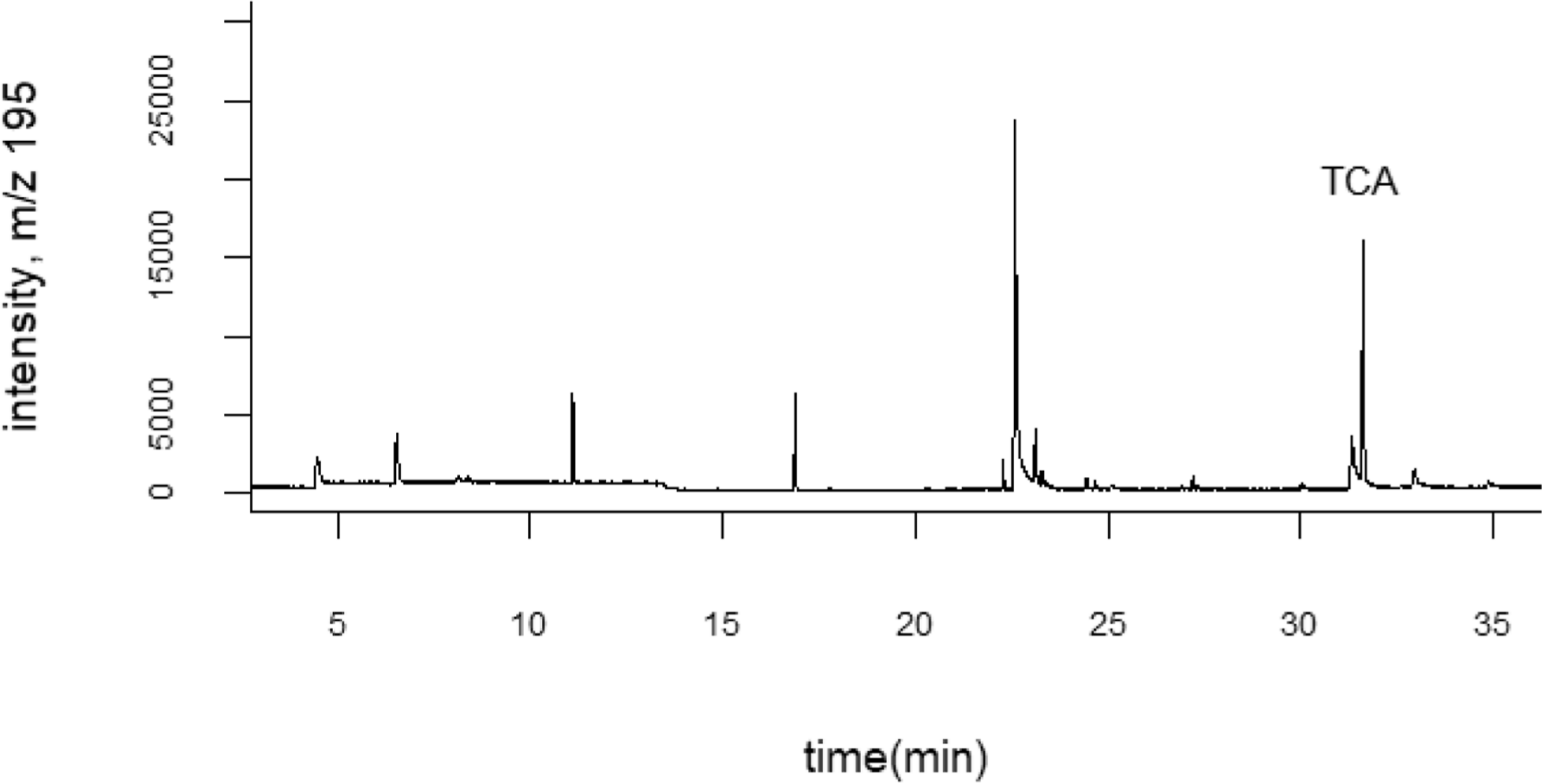
Extracted ion chromatogram (*m*/*z* 195) resulting from the analysis of a Rio defective coffee sample by means of HS-SPME–GC–MS.

TCA determination was also carried out by direct SPME–GC–MS analysis of the headspace of green coffee powder, using an approach alternative to the releasable TCA method and specially adapted to coffee analysis. 5 g of ground green coffee was directly placed in a 20 mL screw-capped vial and kept under stirring at 60 °C. Headspace volatile compounds were extracted for 30 min using a 75 μm SPME fiber coated with CAR/PDMS (Supelco, US). After extraction, the SPME fiber was removed and introduced for 10 min into the injector port of the gas chromatograph at 250 °C. Injection of blanks between samples showed no contamination of the fiber, confirming effective cleaning. GC analyses were performed with an Agilent 7890B gas chromatograph equipped with a 5977B Agilent mass spectrometer (Agilent, US), a PAL RSI 85 autosampler (Agilent, US), and a 60 m ZB-WAX capillary column (film thickness 0.25 μm; internal diameter 0.25 mm, Phenomenex, US). The GC injector was set in the split mode (split ratio of 4:1), and the oven temperature, initially set to 50 °C for 3 min, was then increased to 200 °C at 4 °C/min and then again to a final temperature of 240 °C at a rate of 20 °C/min and held for 5 min. The mass spectrometer was set to the electron impact mode (EI) generated at 70 eV, and mass spectra were collected in the SIM mode using ions *m*/*z* 167, 169, 195, 210, and 212, and the results are expressed in ion *m*/*z* 195 peak area arbitrary units. Analyses were run in duplicate. A representative chromatogram is reported in Figure 2.

## Results and Discussion

The sample set used in this work consisted of 18 batches of “good-quality” green coffee beans from five different geographical origins and 4 Rio defective samples from Brazil (Table 1). The most represented origin was Brazil with eight samples (four “good quality” and four Rio defective samples) since based on what was previously reported,^3^ Brazilian coffees are the most affected by the Rio defect.

**Table 1:** Green Coffee Samples, Type of Analysis Performed, and Results

coffee batch	country of origin	sensory analysis	TCA NO^+^ CI-MS (cps)	releasable TCA (ng/L)^a^	HS-SPME–GC–MS (peak area units)
G1	Guatemala	not defected^b^	n.d.^c^		
C1	Colombia	not defected	n.d.		
B1	Brazil	not defected	n.d.	n.d.	
B2	Brazil	defected	642 ± 19^d^	16.2	49,654
E1	Etiopia	not defected	n.d.		
N1	Nicaragua	not defected	n.d.		
B3	Brazil	defected	105 ± 16	2.4	7,262
G2	Guatemala	not defected	n.d.		
N2	Nicaragua	not defected	n.d.		
B4	Brazil	not defected	n.d.		
E2	Etiopia	not defected	n.d.		
B5	Brazil	not defected	n.d.	n.d.	
N3	Nicaragua	not defected	n.d.		
B6	Brazil	defected	322 ± 24	8.0	24,884
E3	Etiopia	not defected	n.d.		
G3	Guatemala	not defected	n.d.		
B7	Brazil	not defected	n.d.		
G4	Guatemala	not defected	n.d.		
E4	Etiopia	not defected	n.d.		
B8	Brazil	defected	201 ± 22	5.1	13,794
C2	Colombia	not defected	n.d.		
N4	Nicaragua	not defected	n.d.		

a According to ISO 20752:2014.

b Good quality according to illycaffè S.p.A. Quality Control Department (Trieste, Italy).

c n.d. = not detected.

d Mean ± sd, *n* = 3.

**Figure 3:**
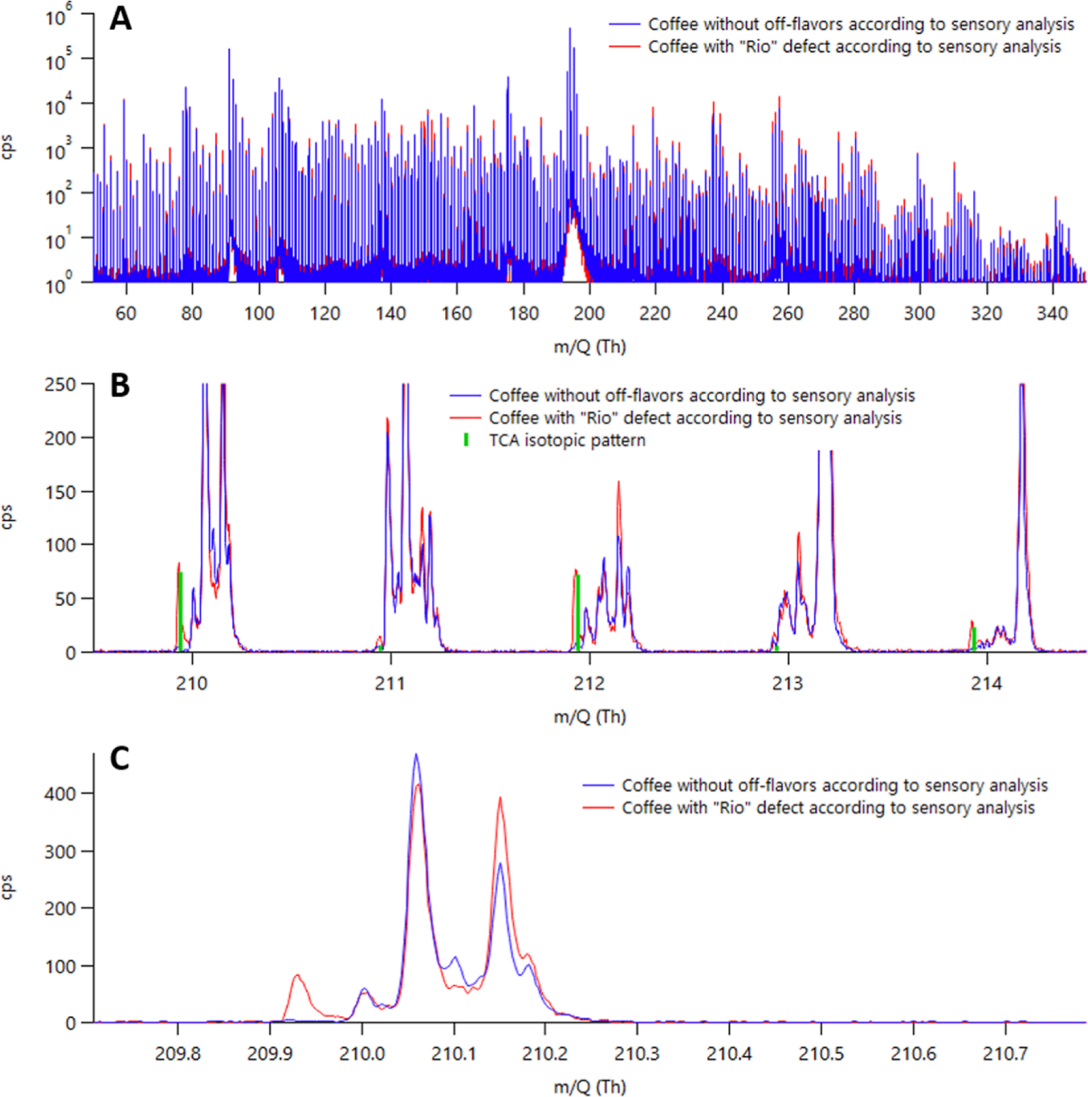
Vocus CI-MS spectra of two green coffee bean powders. 2 s average spectra are reported. A = full spectrum, B = mass range 210–214, and C = detail of nominal mass window *m*/*z* 210.

A correspondence was sought between sensory and instrumental data using Vocus CI-MS. Based on the evidence available so far, it is reasonable to consider TCA to be the key marker for the Rio defect. As demonstrated in the case of cork analysis, it is possible to perform a very rapid (3 s/sample) and direct determination of TCA with a limit of detection below the perception threshold. The same principle was tested replacing the cork with the ground coffee powder. Figure 3 shows representative mass spectra for a defected coffee and a non-defected one. The authentic standard spectrum shows a pattern with very good agreement with what can theoretically be expected considering the natural isotopic abundance of chlorine, which in the case of TCA gives rise to peaks *m*/*z* 209.940 (C_7_H_5_^35^Cl_3_O^+^), *m*/*z* 211.937 (C_7_H_5_^35^Cl_2_^37^ClO^+^), and *m*/*z* 213.934 (C_7_H_5_^35^Cl^37^Cl_2_O^+^). Instead, in the same section of the mass spectrum of the non-defected coffee, no peak can be observed (Figure 3). For the defected coffee, three mass peaks can be found, again showing very good agreement with theoretical results, both in terms of mass accuracy (error < 1 ppm) and isotopic patterns. It is also worth mentioning that in nominal mass windows for *m*/*z* 210, 212, and 214, several potentially interfering mass peaks can be found in the coffee sample headspace: it is therefore extremely important to achieve good mass resolution in order to determine TCA with the good accuracy, sensitivity, and specificity required in this complex matrix. This is made possible using the time-of-flight mass analyzer that achieves a resolving power of *m*/Δ*m* = 15,000. It could still be surmised that, because of the absence of chromatographic separation, under the tested conditions, it is not possible to discriminate between 2,4,6 trichloroanisole and other TCA isomers, but so far, besides 2,4,6-TCP and 2,4,6-TCA, no other chlorophenols or chloroanisoles have been reported for coffee.^3^

The 22 coffees were analyzed in a random order, measuring each coffee batch in triplicate independent samples. In the four defected coffees, TCA was always detected while TCA detection was not possible in any of the “good-quality” non-defected samples. The variability in TCA determination, expressed as standard deviation of the mean obtained on triplicate sample measurements of the defected coffees, was 3–15% (Table 1). Therefore, instrumental determination and sensory determination lead to exactly the same result. One of the advantages of the instrumental method over sensory analysis is that Vocus CI-MS can be quantitative if calibration is carried out.

**Figure 4:**
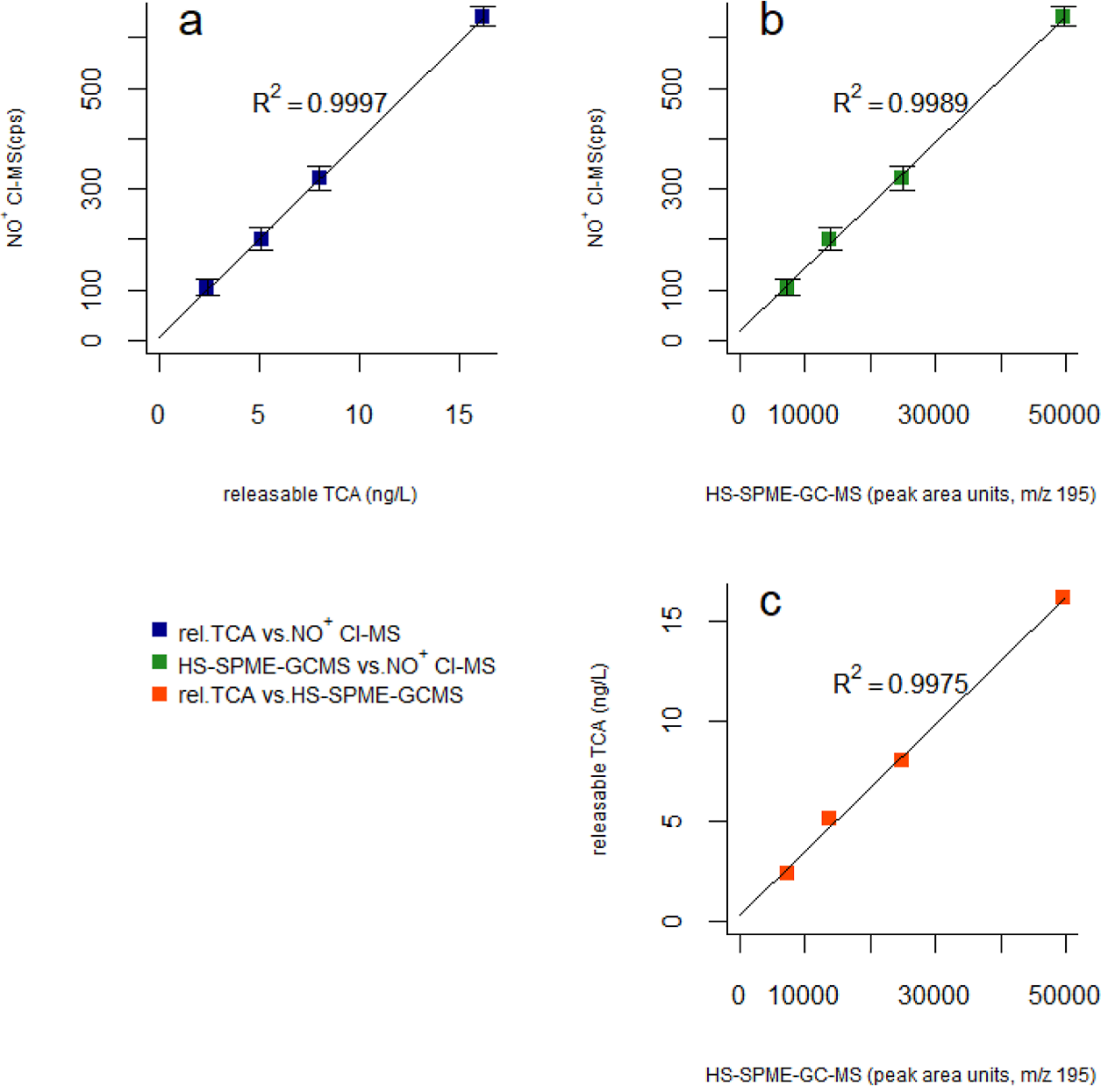
TCA determination in coffees with Rio off-flavor: linear correlation between different instrumental techniques.

Since no reference method is available for TCA in coffee, the releasable TCA method was adapted employing the conditions used for cork granules.^10^ The key literature reference for TCA analysis in coffee adopted a different strategy based on a laborious approach consisting of simultaneous distillation extraction, followed by evaporative concentration and derivatization.^3^ The rationale for the choice adopted in this work was that the releasable TCA method is the industry standard for TCA analysis in cork, where cork taint is a well-known and long-standing problem; it is simpler–however still lengthy—it involves a smaller number of steps and a reduced oxidative stress. It is therefore more likely to produce results that better reflect real conditions. TCA determination was carried out on the green coffee powders according to the adapted releasable TCA method on six selected green coffee samples: the four defected ones plus two non-defected ones, randomly selected from the remaining Brazilian coffee samples. The two non-defected coffees did not contain any detectable TCA, considering that the methodology, based upon SPME–GC–MS, had a detection limit of 0.5 ng/L of releasable TCA. The detection limit for this method was determined to be 0.5 ng/L, considering that the coefficient of variation is lower than 5% for 5 ng/L, when the selected internal standard is the deuterated analogue TCA-*d*_5_ as per criteria specified by the official method. Similar to Vocus CI-MS, GC–MS showed good agreement with sensory analysis for the four defected samples, presenting concentrations of 2.4–16.2 ng/L of releasable TCA (Table 1). The correlation between TCA results expressed as cps as obtained by Vocus CI-MS and releasable TCA levels was outstanding, with a determination coefficient (*R*^2^) of 0.9997 (Figure 4). Good agreement with GC–MS confirms again that, even in the absence of a chromatographic separation, an approach based on direct injection mass spectrometry remains specific for TCA with a remarkable gain in throughput. It also indirectly shows good linearity of Vocus within the assessed range (2–16 ng/L). However, a good linearity extending to up to 5000 ng/L of releasable TCA can be expected, given that a linearity range covering six orders of magnitude has already been shown for the Vocus CI-MS.^16^

An additional comparison is presented using a headspace-SPME–GC–MS approach alternative to the previous one. In this case, the SPME fiber was used to directly sample the headspace of the ground green coffee beans. From the sample preparation standpoint, this solution does not require any solvent extraction, and therefore, it is closer to the approach employed in the case of the Vocus CI-MS. Compared to the releasable TCA method, several differences are present in terms of SPME–GC–MS conditions as each method was specially adapted to the specific type of sample analyzed (green coffee powder vs hydroalcoholic extract). Unlike what was conducted in the releasable TCA method—but similar to the Vocus CI-MS—this methodology is only semi-quantitative, and data are expressed as peak area units of peak *m*/*z* 195 (Table 1), even though calibration and quantitation would be possible if required. Overall, very good agreement was seen with both Vocus CI-MS and releasable TCA with *R*^2^ values equal to 0.9989 and 0.9975, respectively (Figure 4). Even though HS-SPME–GC–MS obviates the lengthy solvent extraction and it can be easily and completely automated, still it can be quite time-consuming with its 30 min headspace equilibration and 40 min chromatographic run. Further improvements in sample throughput for HS-SPME–GC–MS could be achieved by means of method optimization and through the use of narrow-bore chromatographic columns allowing for faster separations.

This work supports the feasibility of TCA determination in coffees by Vocus CI-MS as competitive approach for the rapid detection of the Rio defect. A three-way cross-validation was carried out among three techniques, one of which (the GC–MS analysis using the releasable TCA method) was quantitative. Correlations were excellent in all cases. For the purpose of this work, quantitative aspects are secondary because any detectable amount of TCA renders the product inacceptable for consumption, as highlighted by the comparison between sensory and instrumental data. A possible application of this methodology is the rapid screening in an industrial setting: the extreme rapidity and non-destructive nature of the measurement make this solution a realistic decision-making tool. So far, a limited number of reports are available regarding TCA in coffee and its involvement in the Rio coffee defect.^2−4^ This work extends the scope of TCA analysis in coffee to several samples from different geographical origins, puts into evidence the lack of TCA as a possible “naturally occurring” coffee metabolite in “good quality” samples, and strongly suggests the possible microbiological origin of the Rio defect as previously indicated. The introduction of new quick and sensitive analytical tools could help to provide a more comprehensive picture of the diffusion and relevancy of this coffee off-flavor.
